# Development and application of ribonucleic acid therapy strategies against COVID-19

**DOI:** 10.7150/ijbs.72706

**Published:** 2022-08-01

**Authors:** Lin Ning, Mujiexin Liu, Yushu Gou, Yue Yang, Bifang He, Jian Huang

**Affiliations:** 1 School of Healthcare Technology, Chengdu Neusoft University, Sichuan, China; 2 School of Life Science and Technology, University of Electronic Science and Technology of China, Sichuan, China; 3 Ineye Hospital of Chengdu University of TCM, Sichuan, China; 4 Medical College, Guizhou University, Guizhou, China

**Keywords:** ribonucleic acid therapy, COVID-19, SARS-CoV-2, antisense oligonucleotides, ribozymes, RNA interference

## Abstract

The Coronavirus disease 2019 (COVID-19) pandemic is caused by the severe acute respiratory syndrome 2 coronavirus (SARS-CoV-2), remaining a global health crisis since its outbreak until now. Advanced biotechnology and research findings have revealed many suitable viral and host targets for a wide range of therapeutic strategies. The emerging ribonucleic acid therapy can modulate gene expression by post-transcriptional gene silencing (PTGS) based on Watson-Crick base pairing. RNA therapies, including antisense oligonucleotides (ASO), ribozymes, RNA interference (RNAi), aptamers, etc., were used to treat SARS-CoV whose genome is similar to SARV-CoV-2, and the past experience also applies for the treatment of COVID-19. Several studies against SARS-CoV-2 based on RNA therapeutic strategy have been reported, and a dozen of relevant preclinical or clinical trials are in process globally. RNA therapy has been a very active and important part of COVID-19 treatment. In this review, we focus on the progress of ribonucleic acid therapeutic strategies development and application, discuss corresponding problems and challenges, and suggest new strategies and solutions.

## Introduction

The Coronavirus disease 2019 (COVID-19) is an emerging infectious disease caused by a new form of severe acute respiratory syndrome coronavirus (SARS-CoV), namely SARS-CoV-2 1, 2. As known so far, the SARS-CoV-2 spreads faster but is less pathogenic than SARS-CoV, which affected more than 8000 individuals all over the world with a fatality rate of 11% in 2002-2004 3, 4. Among those affected patients, the failure of lung or related cardiovascular system contributed to a large share of the death. An excessive pro-inflammatory response mediated by elevated inflammatory cytokines and chemokines can lead to lung injury and acute respiratory distress syndrome (ARDS). In addition, viral infection can cause a series of clinical symptoms, such as neurological complications, coagulation disorders, liver and kidney failures as well 5, 6. Since the World Health Organization (WHO) declared the COVID-19 outbreak a global pandemic, as of January 31, 2022, 373,229,380 confirmed cases, including 5,658,702 deaths, have been reported 7, 8. Another disturbing concern is the variability of SARS-CoV-2. From the Alpha variant to the latest Omicron variant, the pathogenicity and transmissibility of the virus are qualitatively changed compared with the original strain, adding to the complexity of and difficulty in the pandemic prevention and treatment 9, 10. To keep COVID-19 at bay, scientists have developed various vaccines to enhance immunity, and accelerated the development of therapeutic drugs, including small molecule and antibody drugs 11-14. Among them, ribonucleic acid therapeutic strategies, which saw rapidly development in the past few years, seem to be most potential in treating COVID-19.

## RNA Therapy

The classic central dogma of molecular biology believes that RNA is the transmitter of genetic information between DNA and proteins 15, and the discovery of catalytic RNA in the 1980s expanded the functions of RNA 16. The Human Genome Project (HGP) and the progress in high-throughput sequencing technology dug up further information on the human genome, especially the gradual identification of the functions of a range of non-coding RNAs, completely changing researchers' perception of the RNA world 17. It is now believed that RNA regulates the genes' functions in all living cells, and is closely related to many diseases 18. In the 1990s, after the discovery of RNA interference (RNAi) 19, RNA-based biotherapeutics came into being. Researchers tried to control gene expression permanently or temporarily by transporting nucleic acids into cells so as to treat the diseases. Thus, the era of RNA therapy has begun.

Under most circumstances, RNA therapies can be classified into several categories, including antisense oligonucleotides (ASO) which inhibit the translation of mRNA, RNAi, aptamers that could bind proteins and other molecular ligands, small activating RNAs (saRNAs) with the ability to activate genes, catalytic RNAs or called ribozymes, RNA vaccines and gene editing approaches such as CRISPR (Clustered Regularly Interspaced Short Palindromic Repeats)-Cas (CRISPR-associated) systems 20. RNA based biotherapeutics have natural advantages over the traditional treatments. Since most RNA therapeutic drugs depend on the principles of Watson-Crick base pairing, it can specifically and closely complement and bind to the target to serve the purpose of treatment 21, 22. Besides, the therapeutic RNAs can be designed to suppress any genes, regardless of whether they are noncoding or highly expressed, and thus have the potential to treat a range of diseases theoretically. What's more, RNA can be synthesized through chemical methods, which is more cost-effective and convenient than other biologics. Therefore, RNA therapeutic drugs have significant potential in application because of their high efficacy, specificity and wide range of application 20.

Over the past two decades, a dozen of RNA therapeutic drugs have been approved by the US Food and Drug Administration (FDA). The specific list can be found in Table 1. Fomivirsen (trade name: Vitravene) is the first antisense oligonucleotide drug approved for the treatment of cytomegalovirus (CMV) retinitis complicated by AIDS patients 23. Antiviral effects are exerted by antisense inhibition of CMV mRNA 24. On August 10, 2018, the world's first small interfering RNA (siRNA) drug Patisiran (trade name: Onpattro) was approved by FDA. It is used to treat hereditary transthyretin-mediated amyloidosis and acute hepatic porphyria (AHP), as the first RNAi based drug in the world 25. In 2021, faced with the growing threat from the COVID-19 pandemic, FDA approved two mRNA vaccines developed respectively by the companies of Pfizer/BioNtech and Moderna 26, 27. So far, over 400 RNA-targeting drug development projects have been conducted worldwide, two-thirds of which are in the pre-investigational new drug (pre-IND) stage, one-third in early clinical trials (phase I or II), about 3% in phase III, and some waiting regulatory approval. As most of the RNA drugs deliver a good performance, such as Patisiran with sales over $150 million in 2019, the world's major pharmaceutical companies have made heavy investment in RNA therapeutic drugs to accelerate their development 28.

Viral infection is a common clinical disease 41, and can be a serious threat to human health as some viruses spread rapidly and have a high fatality rate. Today, there are two major interventions for viral infection: one is vaccines, the other is antiviral drugs to control clinical symptoms and reduce viral activity 42, 43. However, the slow development of vaccines and the lack of specificity for most antiviral drugs make it more than urgent to find new antiviral strategies. The RNA therapeutic drugs may provide us with the answer 44. Taking RNAi as an example, it can silence or suppress the expression of genes related to viral replication or some other important biological functions to fight against viral infection. Many researches on RNA therapy against viral infection are going on, including hepatitis B (HBV) 45, AIDS (HIV) 46 and hand-foot-and-mouth disease related virus 47, etc. In the detection and treatment of coronavirus infection, including SARS-CoV, Middle East Respiratory Syndrome Coronavirus (MERS-CoV) and SARS-CoV-2 raging around the world over the past two years, several studies on RNA therapy have been reported 48, 49. Those in vitro and clinical trials show that RNA therapeutic strategies can successfully inhibit the replication of the viruses, thus become the most potential useful tool in controlling COVID-19 50.

With great progress in RNA therapy, it still has some limitations in practice. Due to its poor stability, RNA can be easily degraded in organisms by ribonuclease (RNase), making the biological half-life too short. The low efficiency of cellular uptake makes it difficult for the drugs to get into the target cells 20. Besides, RNA itself is potentially immunogenic and likely to have off-target effects 51, 52. Efforts to improve the features of natural nucleic acids and develop them into useful drugs are diminished by these factors. To overcome these shortages, various chemical modifications and novel delivery systems have been designed to make RNA drugs more accurate. RNA therapy is a rapidly developing field and has significant potential in fighting against SARS-CoV-2. This review, with a focus on the development and application of ribonucleic acid therapeutic strategies for COVID-19, addresses its problems and challenges along its progress, and puts forward some feasible strategies and solutions.

## Potential Targets for RNA Therapy against COVID-19

**Figure 1:**
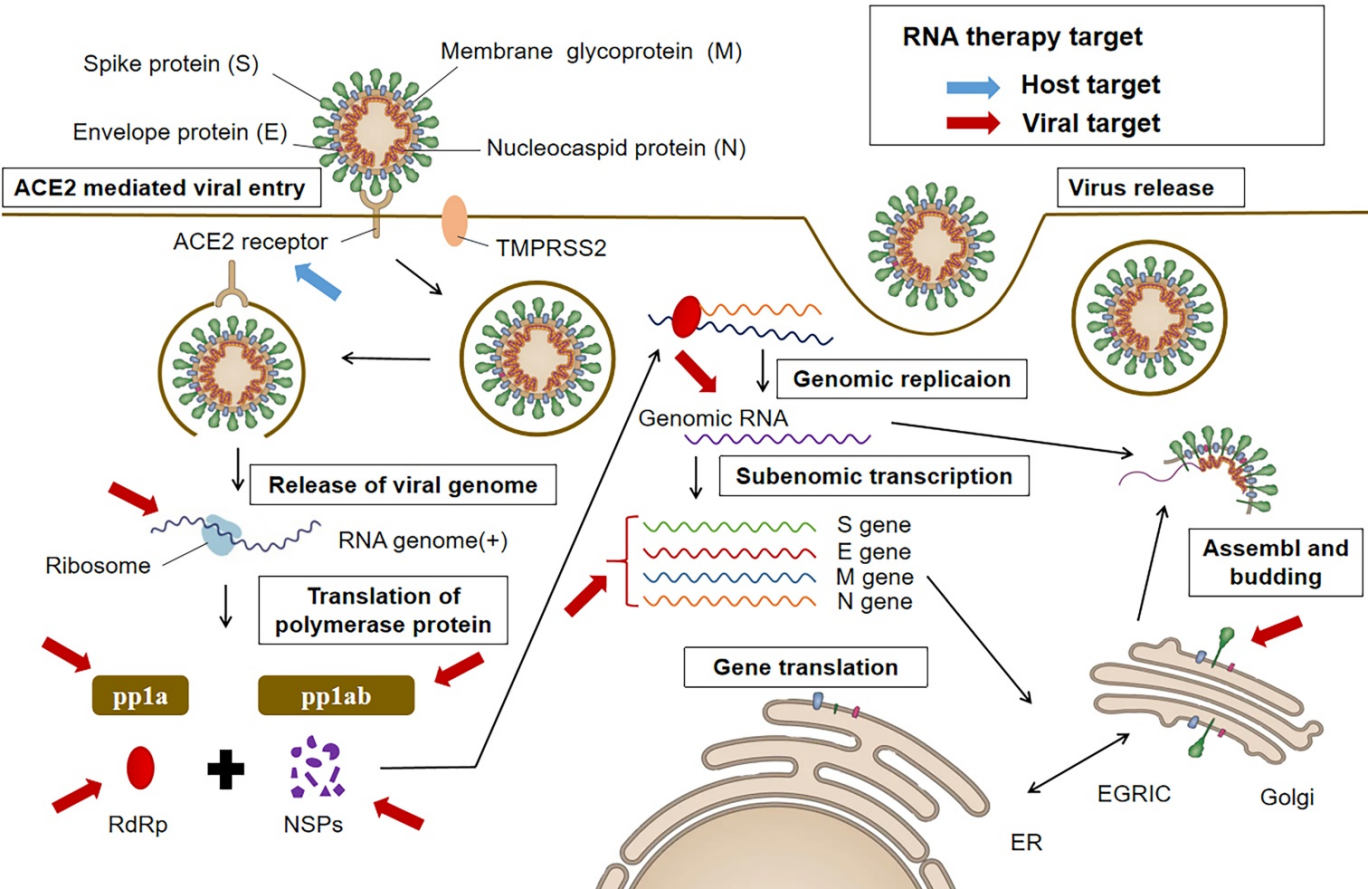
The life cycle of SARS-CoV-2 and potential targets for RNA therapy. All targets can be grouped into two categories. One is the anti-virus (denoted in red), such as the structure genes of viral genome. Another is the anti-host category (denoted in blue), such as ACE2 receptor.

Though being theoretically feasible for RNA therapy to silence or suppress any protein, it is essential to select effective targets for the efficacy of the treatment. In general, there are two antiviral targeting strategies: (i) viral proteins essential in survival and replication, and (ii) related host factors including those involved in cellular entry and trafficking of the virus 51. As shown in Figure 1, RNA therapeutic drugs against COVID-19 can also use the two potential different categories of targets.

### Viral targets

Targeting viral proteins is a direct and effective approach. SARS-CoV-2 has a positive-sense, single-stranded RNA genome, which contains three different categories of genes: structural, nonstructural and accessory 2. There are 4 structural genes including S, E, M, and N, encoding Spike, Envelope, Membrane, and Nucleocapsid protein respectively 53. Among them, S-glycoprotein plays a major role in cell entry via angiotensin-converting enzyme 2 (ACE2) receptor, and so receives the most attention. S protein, with two subunits S1 and S2, determines the viral entry. S1 could directly contact with the host receptor ACE2 with its receptor-binding domain (RBD), while the fusion and entrance of the following membrane to the host cytoplasm of the viruses were mediated by S2 subunit 54-58. Now S protein is the target mostly concerned about for various therapeutic approaches including RNA therapy 12. Other structure proteins of SARS-CoV-2, the M protein plays a central role in viral morphogenesis, assembly and egress 12. Further studies show that the domains formed by the N- and C-terminal of M protein may be the potential RNAi targets 59, 60. The E protein, critical for viral envelope curvature, maturation, and budding, is also involved in ion channel activity that is required for pathogenesis of SARS-CoV and possibly SARS-CoV-2 4. In a study on mutations among 68 samples of SARS-CoV-2, 42 missense mutations in a majority of structural and nonstructural proteins were identified, but the mutations in the E protein were not^61^, indicating that the conserved E protein may serve as a great viral target for RNA therapy. N protein, another viral structural protein, is incorporated into nucleocapsid and responsible for viral genome packing. As its domains can bind to RNA via its phosphorylated residues, the N protein may also be a suitable target 62.

The nonstructural gene ORF1ab encodes polypeptides pp1a and pp1ab (-1 ribosomal frameshift) which occupies about 2/3 of the genome at the 5′ end. Hydrolytic cleavage of pp1a and pp1ab produces a series of nonstructural protein (i.e., nsp1-16) that are essential for viral transcription and replication 53. The productions of accessory genes, although not essential for viral structure or replication, could modulate host innate or adaptive immune response and play a crucial role in viral pathogenicity 63. In theory, all proteins encoded by the SARS-CoV-2 genome are potential anti-virus targets for developing ribonucleic acid therapy strategies against COVID-19.

The untranslated region (UTR) is another important part on the genome of SARS-CoV-2. Several studies show that the 5'UTR and the 3'UTR are also of great significance in the RNA replication and transcription of virus 64. The researcher found in an early study that the small interfering RNA (siRNA) which targeted to the 3`UTR of SARS-CoV could suppress the viral infection and replication in Vero E6 cells 65. Another study showed that the therapeutic siRNA which directly targeted the leader sequence of SARS could also inhibit the viral replication in Vero E6 cells 66, suggesting that the UTR of SARS may serve as a potential target of RNAi. Since the secondary structures of the 5`UTR of SARS-CoV and SARS-CoV-2 are remarkably alike 64, these specific region sequences can also be the target for the treatment of COVID-19 using RNA therapeutics.

## Host targets

As various interactions between the virus and the host proteins can be identified, host targeting is another strategy in antiviral therapy 67-70. The viral infection and replication can be suppressed by blocking or inhibiting the key host factors. The ACE2, serving as the receptor of viral S protein, can facilitate the internalization of SARS-CoV and SARS-CoV-2 71. It has been shown that soluble ACE2 has a protective role in many organs including lungs, making the recombinant ACE2 seem as a suitable therapeutic strategy 72. Subsequent studies pointed out that ACE2 had a paradoxical role in protecting organs and facilitating the viral internalization 73. Thus, further research and discussions on the benefits and potential risks of targeting ACE2 as a therapeutic strategy in COVID-19 are needed 73.

The components of the endocytic pathway have been proposed as crucial targets for developing the therapeutic strategies for all CoV species 74, for the endocytic pathway is thought to be important in viral entry and replication. Chloroquine, with its ability to neutralize lysosomal pH and inhibit protease activity, was extensively used in SARS-CoV 75, 76. It is also reported that Chloroquine and hydroxychloroquine could suppress the viral entry into cells by interfering with the interaction of SARS-CoV-2 with cell surface gangliosides 77. However, the following study manifested that no significant difference was found in the outcome of patients receiving hydroxychloroquine, making this idea remain controversial 78. A recent study showed that AP2-associated protein kinase 1 (AAK1) seemed to be a suitable target to interrupt the viral entry into host cells and its intracellular assembly 79, 80. There are dozens of medically approved AAK1 inhibitors. Among these drugs, Baricitinib, a Janus kinase (JAK) inhibitor, which can bind to cyclin-G-associated kinase along with AAK1 to prevent the entrance of virus into cells and will not cause severe side effects even with higher doses, may be the most potential treatment against COVID-19 81. Another study found that AP2M1 as a crucial host factor in the infection of SARS-CoV and SARS-CoV-2. The specific siRNA, namely siAP2M, was used to knock down the AP2M1 protein involved in endocytosis and significantly suppressed the viral infection 82. These studies demonstrate that the therapeutic strategy of inhibiting the key host factors in endocytosis is feasible.

## Antisense Oligonucleotides (ASO)

The antisense therapeutics was proposed in the 1970s with the theoretical basis that gene expression could be modulated by nucleic acids 83. The chemically synthesized 15-50 nucleotides in length are designed to bind to RNA targets via complementary base pairing for the prevention of mRNA transcription and translation. Transported into cells, ASO combines with the target mRNA, and RNA-DNA complex becomes a substrate for RNase H, leading to endonuclease-mediated transcript knockdown 84. ASO can also directly target mRNA, inhibiting mRNA from 5′ capping, 3′ polyadenylation, and splicing, ultimately terminating protein production 84. ASO therapy is considered to be one of the most effective nucleic acid therapeutic strategies for many diseases, including myopathies, neurodegenerative diseases, oncology, etc.^85^-^87^. Given the advantage of targeting any viral RNA of interest, ASO has already been applied in some highly pathogenic viral infections of Ribovirus, such as Ebola, influenza, Hepatite-C, Japanese Encephalitis, achieving certain efficacy 88-91. It is also an early nucleic acid therapy with successful clinical application for its more potential action mechanisms, easier intracellular delivery, and stable clinical suitability. Fomivirsen (trade name: Vitravene), the first ASO drug, was approved by FDA in 1998 24. After that, 9 ASO drugs were approved (Table 1), and 50 more ASO candidates are in clinical trials 28, 92.

Studies on ASO antiviral therapy against SARS-CoV boomed following the SARS outbreak in 2003. ASO technologies have been advanced to reduce the severity of viral infection, and to diagnose and treat SARS-associated disease. Three ASO-related patents were published. Patent US20030224353 published by Stein David described ASO ORF1 which targeted region 217-245bp of SARS-CoV, to treat the ssRNA viral infection 93. Patent WO2005013905 was filed by AVI BioPharma, Inc., which targeted 3′ terminal end of negative strand using the modified oligonucleotide compounds to treat ssRNA viral infection, including flavivirus, coronavirus, picornavirus, togavirus, calicivirus, and hepatitis E virus 94. Ionis Pharmaceuticals designed hybrid DNA/RNA ASO for the disruption of the pseudoknot in the frameshift site of SARS-CoV to reduce the viral activity. Based on this ASO, Ionis Pharmaceuticals published the patent application WO2005023083 95. In addition to the patents, another study on ASO to target the conserved RNA elements that were required in the synthesis and translation of viral RNA, demonstrated that ASO could effectively inhibit the replication of SARS-CoV 96. However, there appeared some unexpected results that SARS-CoV developed ASO drug resistance and escaped. Therefore, more rationality and caution are needed in designing ASO drugs for coronavirus.

Since the SARS-CoV-2 and SARS-CoV share 79.5% similarity in genome 80, ASO should also be capable of treating COVID-19 on a theoretical level. ASO drug can be designed to bind to the viral mRNA transcripts that encode the crucial proteins associated with the replication and transcription to disturb the viral expansion. Based on this hypothesis, in a recent study, nine ASO candidates were designed to target the genes of viral structure protein N and ORF1a, ORF1b and 5′-UTR of viral genome, of which were very important for the transcription and replication of SARS-CoV-2. The results showed that viral infection could be inhibited by blocking these key targets effectively, which needs further verifications 97. Beyond targeting the important viral protein synthesis, another strategy is to directly target the viral genome itself, especially RNA viruses (other than retroviruses). ASO can be designed to target the conserved sequences in viral genome to disturb or completely degrade the genomic RNA for the elimination of SARS-CoV-2 52. However, more studies are needed due to the unexpected variability of RNA viruses, and the difficulty to find suitable conserved sequences of viral genome for ASO targeting. All in all, with its simplicity to design, low toxicity effect, high specificity, and low production cost, ASO therapy is still a very potential treatment for COVID-19 98.

## Ribozymes

The discovery of catalytic RNA has dramatically complemented and improved the understanding of RNA in the 1980s. Catalytic RNA, also known as Ribozymes, can bind and cleave phosphodiester in nucleic acid to induce specific degradation of target mRNA for the inhibition of gene expression 99. Being natural but also able to chemically synthesized, ribozymes are believed to treat viral infectious diseases effectively for its gene editing activities. Meanwhile, more efforts are needed in the design of ribozymes. The target region is required to be quite conserved and critical for the survival or replication of the virus, and accessible so that the ribozymes can bind to it easily and smoothly 99. With its weak RNase resistance, Ribozymes often perform a short-term effect in vivo 100, 101. Therefore, chemical modification and appropriate delivery system are always added to increase therapy robustness. Another concern is that the cleavage effects in vivo are often different from those in vitro 102. Despite these disadvantages, ribozymes are still regarded as an important and effective antiviral therapy. Compared with some other RNA drugs, ribozymes have high specificity and low immunogenicity 103. Ribozyme-based studies have already been carried out in a series of RNA viruses including HCV, HIV, and influenza 104-107, but few reports about the ribozyme therapy on coronavirus-induced diseases. This may be caused by the fact that ribozymes require for the conserved regions of the genome and the coronavirus families are prone to new mutations, making the design relatively difficult. In addition, in the last two decades, except for SARS-CoV and MERS-CoV, which caused small-scale viral infections in certain areas, coronaviruses did not cause large-scale epidemics until the outbreak of COVID-19 in 2020. In an early study, a therapeutic RNA/DNA chimeric ribozyme was designed to recognize and cleave the conserved common regions and regions with loop structures in genome of coronavirus, including SARS, and a patent application was applied in this research (JP2007043942)^108^. As the SARS-CoV-2 and SARS-CoV share highly similar genome 80, this designed chimeric ribozyme may also be potentially useful for COVID-19's treatment and related studies.

In recent years, gene-editing technology represented by CRISPR/Cas system is on a rise. Some studies have tried to use gene therapy technologies in combination with ribozymes to modulate gene expression so as to treat some intractable diseases. Ribozymes in conjunction with Cas9, or even Cpf1 nuclease are believed to improve the efficiency of treatment strategies 109. CRISPR-RGP, a gene-editing system based on *Streptococcus pyogenes* (Sp) Cas9, was designed against Plasmodium. A ribozyme-guide-ribozyme (RGR) single guide RNA (sgRNA) expression strategy with RNA polymerase II promoters were utilized in the system, and a position-dependent but strand-independent reduction in gene expression was induced by the catalytically dead SpCas9 (dSpCas9) binding to the upstream region of target genes. This robust gene-editing system can be used in the treatment of malaria by generating gene disruptions and insertions of coding sequences in Plasmodium 110. Similar studies with other gene therapy technologies on HIV have been carried out 111. Therefore, gene editing or gene therapy combined with ribozymes provides a possibility to treat SARS-CoV-2 infection.

## RNA Interference (RNAi)

**Figure 2:**
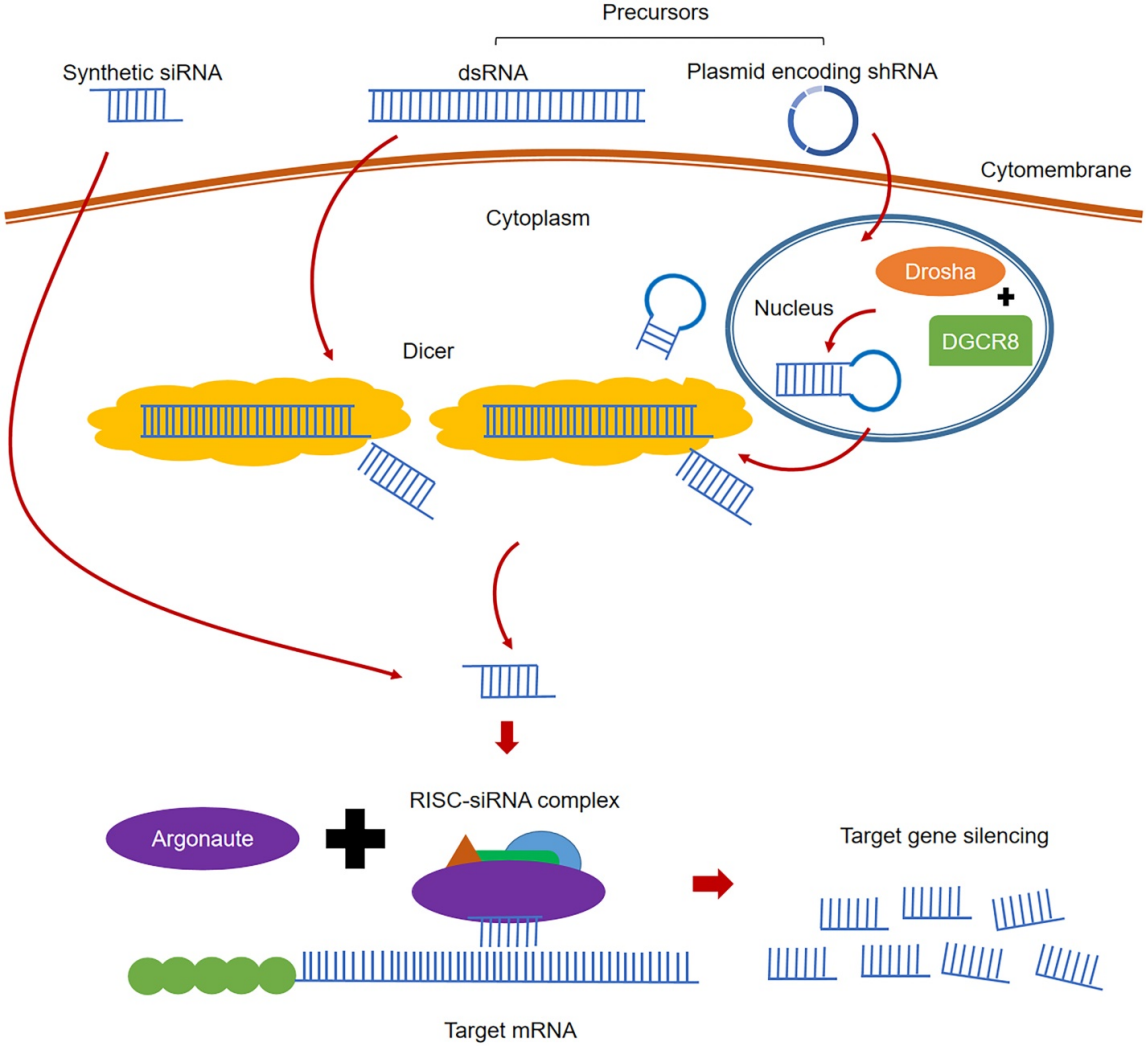
The classical processes of RNAi.

RNA interference (RNAi), the post-transcriptional gene silencing (PTGS) phenomenon, is induced by double strand RNA (dsRNA) which could efficiently and specifically degrade the homologous mRNAs 19. RNAi is an inherent habitant of living organisms, which can be triggered by small interfering RNA (siRNA) or dsRNA. The classical process of RNAi is shown in Figure 2. In the cytoplasm, the long pieces of dsRNA are firstly cleaved into siRNA (21-23 nt in length) fragments by the enzyme Dicer 112. Unlike ribozymes or ASOs that directly bind to target RNA, siRNA is combined with the protein Argonaute (Ago) and forms a complex called RNA-induced silencing complex (RISC) 113. With siRNA being unwound, the activated RISC complex containing the antisense strand (or guide strand) of siRNA will specifically target and degrade the complementary mRNA 114. Two methods are used to artificially induce RNAi using siRNA strategy: directly delivering the designed siRNA into target cells via appropriate transporters, or using designed plasmids encoding short hairpin RNA (shRNA). When the plasmid is sent into target cells, shRNA is produced and cleaved by Drosha and DGCR8 in the nucleus. After transferring into the cytoplasm by exportin 5, shRNA can be cleaved by enzyme Dicer and turn into siRNAs to induce RNAi 115. Since siRNA can suppress gene expression by the RISC mechanism, target genes will see continuous degradation, resulting in extremely high potency and sustained activities compared with other RNA therapies. RNAi requires extremely strict recognition to degrade specific sequences, making its specificity highly unparalleled. In addition, small doses of siRNA could trigger sustained gene inhibition, and the effect increase with its concentration level 115. Based on these advantages of gene modulation, RNAi technology has been widely used to explore gene function and in the treatment of infectious diseases and malignant tumors 116-120. Currently, there are four siRNA-based drugs approved (Table 1) 25, 37, 39, 121, and more than 20 candidates in clinical trials 28.

## Past experience of RNAi against SARS-CoV

RNAi technology was applied in the previous treatment research for the SARS epidemic. SARS-CoV could enter human cells through the S protein binding to the host receptor ACE2, which is similar to SARS-CoV-2. Therefore, S protein has become an important target selection in RNAi therapy. Several studies demonstrated that the viral load can be effectively reduced by silencing the S protein using siRNA strategy 65, 122, 123. N protein incorporated into nucleocapsid and responsible for viral genome packing is also considered as a viral suppressor of RNAi in host cells 124. The studies of targeting gene encoding N protein using shRNA approach were also carried out, with effective reduction in viral loads 125-127. An interesting finding in the studies is that inhibition of N-gene could lead to an increase in the secretion of INF-β, which also can help cells to fight viral infection. Apart from S and N protein, another structure protein M of SARS-CoV is also explored. A former study concluded that targeting the 3` portion of gene encoding M protein could effectively cut the target mRNA levels, but left the inhibition of SARS-CoV uninvestigated 128.

In addition to structural genes, genes encoding nonstructural proteins and accessory proteins and the UTR regions are also important targets of RNAi therapy. The RNA-dependent RNA polymerase (RdRp) gene was highly conserved in different coronaviruses and considered to be an effective target 129. Studies using siRNA and shRNA showed effective silencing of gene encoding RdRp^130^-^132^. However, the RdRp gene appeared to have different sensitivity to different siRNA sequences, which required more caution and rationality in the design of siRNA. In a separate study, shRNA was designed to target a leader sequence thought to be conserved in all coronaviruses, and a reduction in the viral load was observed 133. Another study focused on nsp1, a non-structural protein of SARS-CoV which was derived from the 5′ leader end of the genome. The cells transfected with the designed shRNA can resist the viral invasion and enjoy adequate protection 134. The subgenomic RNAs (sgRNAs), which are generated from discontinuous transcription during the synthesis of negative-strand genomic RNA of coronavirus, are also of great importance. The translation of most viral genes (the structural and accessory proteins) occurs via sgRNAs as the intermediates 135. In a past study, several accessory proteins derived from the sgRNAs of SARS-CoV were considered to have no significant sequence homology compared with other coronaviruses. The previous experiments demonstrated that these proteins were suitable and effective targets as well as the S protein 136.

## RNAi therapy against SARS-CoV-2

Since the SARS-CoV-2 is 79% similar to the SARS-CoV in genome, theoretically, RNAi strategies for SARS should be effective in treating COVID-19 as well 137. siRNA can be designed to target the structural genes including S, E, M and N proteins to inhibit the entrance of virus into host cells and disturb the viral replication 138. Besides the structural genes, the leader sequence seems to be another suitable choice 133. The experience of SARS-related research can assist the development of therapeutic siRNA targeting leader sequences to inhibit the replication of SARS-CoV-2. In a recent study, eight siRNAs targeting the highly conserved 5`-UTR of SARS-CoV-2 were designed. siCOV6, the most promising candidate, could reduce the viral replication and prevent cytopathic effect from developing by targeting the leader sequence that was present in all sgRNAs 139. Targeting Nsps is another important strategy for the effective inhibition of viral replication in reducing the viral symptoms 134. There are totally 15 nsps, nsp1 to nsp10 and nsp12 to nsp16, which are encoded by genes on ORF1ab and ORF1a in the genome of SARS-CoV-2. Nsp12 is the RdRp of the virus and can co-regulate the viral replication with nsp5 (3C-like protease) 138. Several previous studies showed the effective suppression of SARS-CoV by using siRNA to target RdRp gene, and this strategy may also be feasible for SARS-CoV-2. In a recent study, nsp9 and nsp10 were found to interact with NF-κB-repressing factor (NKFR) to facilitate interleukin-8 (IL-8) induction in lung epithelial A549 cells. The designed siRNAs were used to target these two nsps, and the introduction of IL-8 was observed to decrease effectively, which could help to reduce the level of inflammation and minimize damages to the lung 69.

## The design of RNAi using computational approaches

In the past decade, with the rapid development of artificial intelligence and the continuous accumulation of biological data, computer technology plays an increasingly important role in drug design. Compared with traditional method of small molecule drug screening, the computational algorithms represented by deep learning can remarkably shorten the development time and cut the cost. In research to fight SARS-CoV-2, due to the time-consuming experimental methods, computational approaches can focus on thousands of solutions simultaneously, and effectively narrow down the cases for experimental validation. Several studies on the RNAi therapy against COVID-19 through the computational methods were reported (Seen in Supplementary Table 1). In one research project, genes encoding nucleocapsid phosphoprotein and surface glycoprotein of SARS-CoV-2 were chosen as the targets, and 8 siRNA molecules selected from 78 candidates were predicted to effectively silence the target genes for the antiviral treatment 140. Nucleocapsid is encoded by N gene and responsible for viral encapsidation and genome packing. In one attempt, several bioinformatic tools were applied for the design of a duplex siRNA molecule that did not fit any off-target sequences. The results demonstrated that it could effectively silence the N gene, with therapeutic potential for SARS-CoV-2 141. In another study, the online software siDirect version 2.0 was used to predict the highly effective siRNAs taking the leader sequence of viral genome as the target, and the HNADOCK online server was applied in molecular docking to validate siRNA affinity for the targets. Four potential siRNAs were revealed in the results. Among them, the siRNA with sequence 5'GUUUAGAGAACAGAUCUACAA3' possesses the greatest potential to inhibit the leader sequence of SARS-CoV-2 with least off-target effect 48. ORF1ab, the largest gene in the genome of SARS-CoV-2, encodes polyprotein PP1ab and PP1a which are the key factors in viral transcription and replication. In a separate study, some bioinformatic approaches were used to predict the potential siRNA by screening siRNA library targeting the ORF1ab gene. In the results, a total of 10 siRNA candidates were sorted out as the potential therapeutic agents against COVID-19 142. With RdRp as another important potential target for RNAi therapy, a dozen of siRNAs were designed to silence RdRp gene to cure COVID-19 in a recent study. Out of the expectation, the target of designed siRNA revealed no significantly matches with the entire human genome, which excludes any possibility for off-target silencing of the siRNA 49. In addition to these structural or non-structural genes, the viral genome can also be targeted by siRNA. In one attempt, siRNAs were designed to directly target the evolutionarily conserved regions in the SARS-CoV-2 genome to down-regulate or silence its RNA. These siRNA candidates which had high affinity and low side effects were considered to be the potential therapeutics 143.

## Industry applications on RNAi therapy against coronavirus

Since the SARS epidemic, dozens of patents for RNA-based therapeutic strategies have been issued. Most of them are based on RNAi therapy, and some are treated with antisense RNA. The review of siRNA-based patents issued or under consideration for coronaviruses is shown in Table 2. Due to the limited number of infected patients and areas of SARS and MERS, and the fact that similar viral infection incidents did not occur after the end of the epidemic in a short run, these treatment patents for coronaviruses were rarely developed into drugs or therapeutic strategies 4. As a result, after the outbreak of COVID-19, there is still no specific cure for SRAS-CoV-2. However, with the advantages of RNAi technology in treating viral diseases and the research experience of RNA therapy against SARS-CoV, some pharmaceutical companies have published their recent research achievements. Hundreds of siRNAs have been designed to target the highly conserved regions of SARS-CoV-2 or the key factors that are responsible for the viral transcription or replication together with the delivery systems. With the ongoing epidemic of COVID-19, these siRNAs could eventually be developed into specific drugs for clinical application.

## Other RNA Therapy

### Aptamer

Aptamers, single-stranded RNA or DNA molecules, can fold and form stable three-dimensional structures by complementary base pairing, electrostatic interactions, and hydrogen bonding, and bind to target proteins with high specificity to modulate the functions 144-146. Although most of aptamers are based on DNA molecules, for the significant advantages of high affinity, facile chemical modification, low-cost, low-temperature sensitivity, rapid synthesis, and large-scale production, RNA aptamers have also been widely used, especially in some antiviral studies^147^-^149^. In this review, we focused on the RNA aptamers against coronavirus infection. Nowadays, aptamers are often combined with other therapeutic agents, such as siRNA and ribozymes 145. In a recent study, siRNA technology combined with aptamers have been applied to suppress HIV-1 expression 150. Researches on aptamers against SARS-CoV have also been reported. In two Korea patent applications (KR2009128837 and KR2012139512), aptamer technology was used to inhibit the SARS virus, which all provide a new RNA therapeutic strategy for the fight against SARS-CoV-2 108.

Aptamers also have a wide application in diagnosis for their high affinity to bind to target proteins. During the SARS epidemic, aptamer technology was proved to be useful in the diagnosis of SARS-CoV. By targeting antigenic viral proteins, aptamers were also considered promising in the diagnosis of COVID-19 151, 152. On this subject, several studies have stated that the aptamer technology combined with the enzyme-linked oligonucleotide assay to diagnose COVID-19 disease are being developed by Aptamer Group 153, 154. Pinpoint Science has proposed a new approach for rapid detection of SARS-CoV-2 by an aptamer-based nanosensor 155. With the progress in aptamer technology, a future where COVID-19 can be diagnosed at speed and with accuracy is foreseeable.

### Peptide nucleic acids (PNAs)

PNA is a specific kind of antisense therapy, in which N-(2-aminoethyl) glycine units has replaced the nucleic acid phosphodiester backbone via a methyl carbonyl binder 156. Similar to ASO, PNAs can specifically bind to target DNA or RNA according to Watson-Crick base pairing to modulate gene expression 157. PNAs can exist stably in vivo or in vitro as the result of the resistance to nucleases or proteases, and have low toxicity due to low or no binding affinity to serum proteins 158, making them important candidates for antisense therapeutics. In some earlier anti-HIV studies, PNAs molecules were designed to target certain regions, including gag gene, dimerization initiation site and att sequences of linear pro-viral DNA's U3 and U5 region. The results demonstrated that PNAs could effectively inhibit the reverse transcription and prevent the integration of the viral genome and the host genome, showing a great antiviral effect 159-161.

The studies on PNAs against coronaviruses is limited. -1 Ribosomal frameshifting (PRF) hardly occurs during translation naturally. However, it can be triggered by specific signals which increases the possibility of tRNA slippage up to 50% 162. The RPF signal consists of two elements, a heptanucleotide slippery site and a downstream tertiary RNA structure in the form of an RNA pseudoknot. SARS-CoV initiates -1 RPF at the three-helix-containing RNA pseudoknot 163. The control of -1 PRF efficiency has been shown to be critical for the maintenance of correct stoichiometric ratios of viral replicase proteins 164. In a previous study, a fully compatible sequence-specific PNA was designed to block the -1 PRF signal which was in charge of the synthesis of viral RNA replicase polyproteins, and the viral replication was significantly inhibited 165. Since the structure of the -1 PRF signal is extremely conserved and stable, the result is also helpful for the design of PNAs against COVID-19. Another problem affecting the PNAs strategy for the treatment is the slow cellular uptake of PNAs 157. Therefore, necessary modifications or appropriate delivery system such as nanotechnological tools should have been applied in the design of PNAs.

### mRNA encoding therapeutic antibodies

Neutralizing antibodies are important biological products against various infectious viral pathogen. Compared with the traditional production and purification processes of therapeutic antibodies, delivering the genetic information of the antibody including DNA or mRNA to produce biologicals in situ is a cost- and labor-effective manner and a new antiviral option 166. In early attempts, naked plasmid DNA (pDNA) was injected into the quadriceps of mice and the local expression of the encoded proteins was observed 167. However, considering the potential integration of the pDNA into the host genome and the fear of anti-DNA autoantibodies, there is no pDNA-based drug been marketed so far even if several pDNA-encoded antibodies were evaluated in phase II-III clinical trials. In recent years, with the progress of in vitro transcribed (IVT) mRNA technology, it has become possible to use engineered mRNA to generate therapeutic antibodies in vivo. IVT mRNA can efficiently induce protein expression, avoiding risks such as be integrated into the host genome. With the improvement of delivery systems, the stability and translatability of the engineered mRNA have also been greatly increased. mRNA therapeutics has been applied in several bio-medical fields including the therapeutic cancer vaccine 168, 169, prophylactic vaccine 170, 171 and different infectious diseases 172, 173.

In a study treating respiratory viral infections, the engineered mRNA encoding a bispecific single-domain antibody against the influenza A virus was constructed and delivered into lungs of mice before the viral infection. The reduced viral titers and morbidities in mice were observed 174. In addition, mRNA therapeutic strategy has also been applied in the treatment against COVID-19. A safe and cost-effective platform to express neutralizing antibody in the lung with replicating mRNA using alphavirus replicon particle (VRP) delivery system is developed. The replicating mRNA encoding therapeutic antibody is intranasally delivered to target lung of mice. The experiment result shows that SARS-CoV-2 can efficiently be suppressed with reducing viral titer and less tissue damage 175. Although there are still some concerns such as the intrinsic immunogenicity and transmission efficiency, a growing number of studies have shown that the local delivery of mRNA encoding therapeutic antibodies in the lungs could be a promising pulmonary antiviral prophylactic treatment 176.

### CRISPR/Cas technology

CRISPR (Clustered Regularly Interspaced Short Palindromic Repeats)-Cas (CRISPR-associated) systems have greatly improved our ability to edit genes and modulate gene expression 177. Except for its genome-editing ability, the diagnostic potential was also noticed by scientists. In 2017, Feng Zhang's group have reported SHERLOCK, the first CRISPR-based nucleic acid detection technique 178. SHERLOCK allows multiplexed, portable, and ultra-sensitive detection of RNA and DNA from clinical samples. Recently, CRISPR/Cas technology has also been applied in the diagnosis of COVID-19. An assay design tool and a research method based on the CRISPR-Cas13 was designed. It was reported that the system could detect specifically SARS-CoV-2 and 66 related viruses in patient specimens 179.

In addition to the diagnosis, CRISPR/Cas technology has already been used in the treatment of SARS-CoV-2. PAC-MAN (prophylactic antiviral CRISPR in human cells), based on CRISPR-Cas13 technology, is found to effectively degrade the SARS-CoV-2 sequences in human lung epithelial cells. In this system, the CRISPR-derived RNAs (crRNAs) were designed and screened, and the crRNAs could target the conserved viral regions to cleave SARS-CoV-2 genome 180. There are totally 22 crRNA pools analyzed by bioinformatics tools which can target all sequenced coronaviruses in PAC-MAN system in this study. Thus, PAC-MAN is a promising strategy to combat coronavirus by targeting different regions of a virus or different coronavirus strains simultaneously with the crRNA pool, preventing possible viral escapes 180.

### Challenges and Perspectives

The right target selection is the first step in RNA therapy against COVID-19, whether ASOs or RNAi strategy. The targets should be conserved in the viral genome and very important for the life cycle of the virus. Similar to other treatments such as therapeutic antibodies, the structural genes of SARS-CoV-2 genome which encode S, E, M and N proteins, as well as crucial NSPs and accessory proteins are all potential therapeutic targets 12. Among them, S protein with the function to help viruses enter cells by binding the host ACE2 receptor has attracted much more attention. Almost all RNA therapies have tried to target S protein to suppress the SARS-CoV-2 55, 60, 123. In addition, proteins responsible for the important viral physiological functions, such as transcription and replication, taking RdRp as an example, are often selected as therapeutic targets too 49. One concern for the treatment is the variability of coronavirus. At present, dozens of new variants of SARS-CoV-2 have been discovered, posting serious challenges to its diagnosis and treatment 10. Therefore, further in-depth research is needed to explore the conserved or specific functional regions suitable for therapeutic targeting in the viral genome by comparing the differences between variants. Another therapeutic strategy is the host targeting, such as targeting ACE2 receptors or some other key host factors in the process of viral transcription or replication 73. However, the suppression of these host genes might cause different unexpected symptoms in humans. The design of RNA therapeutic strategy against host targets should be more cautious.

Another challenge is the stability of RNA-related drugs. It is well known that RNA has weak resistance to nucleases, and is rapidly metabolized in cells. These disadvantages have significantly limited the development of nucleic acid drugs. To solve these problems, several biological techniques, including chemical modifications to the RNA as well as drug delivery system, have been applied in drug design. A wide variety of chemical modifications, including modifications in nucleobase, backbone and sugar moiety, have been studied to improve the affinity and nuclease resistance of the RNA therapeutics 181-185. The therapeutic efficacy of RNA drugs can be considerably enhanced by the chemical modifications through improving pharmacokinetic characteristics, making it a general and common approach for the design of nucleic acid strategy. In addition to the chemical modifications, the drug delivery system is another probable approach. Drug delivery system or the carrier technology can help drugs be taken up by cells more efficiently, reach the therapeutic targets more directly, enhance the targeting effect and biological activity, and prevent the drugs from being degraded by nucleases 186. Liposomes, cationic polymer complexes, dendrimers, polymer microsomes, peptide protein delivery vehicles, and nanoparticles are all thought to be suitable choices of delivery systems 20. Among them, nanostructure lipid carrier (NLC) is considered the most promising drug delivery system at present. Owing to the suitable volume, high drug loading efficiency and specific physical and chemical properties, the NLC system can improve the stability of nucleic acid drugs, complete drug delivery, release the drug effectively, and avoid the drug degradation in vivo. NLC technology has already been applied in Patisiran, the first approved siRNA-based drug 25. Therapeutic siRNA is encapsulated in lipid-based nanoparticles to prevent the degradation by exonuclease while facilitating the transport process in vivo, which is also applicable to the development of nucleic acid drugs for coronaviruses. Because of the acute lung injury and inflammatory response caused by SARS-CoV-2, RNA therapeutic drugs cannot get through the barriers composed of the intense mucus layer, alveolar macrophages and pulmonary proteases 187. Therefore, effective chemical modifications and drug delivery system such as NLC will boost the development of RNA therapeutic strategies against COVID-19.

How to deliver RNA therapeutic drugs to the lungs is another concern. Inhalational delivery, verified by accumulating evidence, is the optimal drug delivery approach compared to some conventional methods. Oral, nasal, and other inhalation delivery have several advantages, such as the ease of administration and the rapid onset of action, which is already used in some drugs and vaccines 188, 189. In 2003, the live attenuated influenza vaccines in the form of nasal spray were approved in US for its better mucosal immune responses 190. Inhalation delivery, or pulmonary delivery, is commonly applied in respiratory diseases. Drops, liquid sprays, powder sprays or gels are the main forms in the treatment. In recent years, nanoparticle formulations, with much garnered attention in the development of RNA therapy because of their ability to carry nucleic acid drugs, can also be used for inhalational delivery. In one RNAi study of lung cancer, therapeutic siRNA was encapsulated in liposomes and then delivered to lungs as spray freeze-dried nanoparticles for treatment 191. Other studies on nanoparticle formulations for pulmonary delivery, such as dry powder chitosan nanoparticles and nanoparticles with the pulmonary surfactant Curosurf, showed that both can deliver therapeutic RNA molecules to lungs through inhalation 192, 193. In a recent exploration, a novel lipid nanoparticle (LNP) delivery system is developed and therapeutic siRNAs which are encapsulated in the LNPs are transported to lungs directly. By using LNP system, SARS-CoV-2 can be greatly inhibited by screened siRNAs which can target the highly conserved regions of the viral genome 50. Another interesting finding was that siRNA formulated with the traditional polymeric carrier polyethyleneimine (PEI) appeared to have the same therapeutic effect as the siRNA without a carrier. The result suggested that inhalation approach might be able to maintain the activity of "free" siRNA molecules 194. Therefore, inhalational approaches serve as a reliable delivery strategy of great significance in winning the battle against SARS-CoV-2 using a nanoparticle delivery system or the “free” therapeutic RNA molecules.

Still there are some pharmacodynamic-related challenges on RNA therapy, including off-target effects and potential immunotoxicity. Some biological technologies such as chemical modifications can hold the key to solutions. However, a more directed effort is required by experts and funding agencies to develop simple, cost-effective, and easy-to-use formulations of RNA therapeutics, to accelerate the clinical application of RNA-based antiviral drugs.

## Conclusion

The outbreak of COVID-19, a particularly contagious respiratory disease caused by the novel coronavirus SARS-CoV-2, has highlighted the urgent need for new therapeutic strategies that are rapid, precise, stable, and target-specific for treatment. In this review, we summarized the research experience of ribonucleic acid therapy strategies against SARS-CoV-2, including ASO, ribozymes, RNAi technology, aptamer, etc., and described different RNA therapeutics in terms of mechanisms, application methods, and shortages to improve. Besides, it paid special attention on the computational approaches in RNAi technology for COVID-19. RNA therapeutic strategies in this review aimed at viral therapy for SARS-CoV-2, provide sufficient evidence of their potential. In the end, from the perspectives of the existing literature, available technical tools, a large number of relevant scientific studies and persistent clinical trials, it is believed that more novel RNA therapeutic drugs will emerge for diagnosis, treatment and prevention of COVID-19 in the near future.

## Supplementary Material

Supplementary table 1.

## Figures and Tables

**Table 1:** The approved RNA therapeutic drugs and vaccines

Categories	Drugs (Trade name)	Indications	Years	Locations	Developers	Reference
ASO	Fomivirsen(Vitravene)	CMV	1998	US	lonis/Novartis	24
ASO	Mipomersen(Kynamro)	HoFH	2013	US	lonis/Sanofi	29
ASO	Eteplirsen(Exondys 51)	DMD	2016	US	Sarepta Therapeutics	30
ASO	Nusinersen(Spinraza)	SAM	2016	US	lonis/Biogen	31
ASO	Inotersen(Tegsedi)	hATTR	2018	US	lonis	32
ASO	Volanesorsen(Waylivra)	FCS	2019	EU	lonis	33
ASO	Golodirsen(Vyondy 53)	DMD	2019	US	Sarepta Therapeutics	34
ASO	Viltolarsen(Viltepso)	DMD	2020	US/Japan	Nippon Shinyaku	35
ASO	Casimersen(AMONDYS 45)	DMD	2021	US	Sarepta Therapeutics	36
siRNA	Patisiran(Onpattro)	FAP	2018	US/EU	Alynlam	25
siRNA	Givosiran(Givlaari)	AHP	2019	US	Alynlam	37
siRNA	Lumasiran(OXLUMO)	PH1	2020	US/EU	Alynlam	38
siRNA	Leqvio(Inclisiran)	Primary hypercholesterolaemia/mixed dyslipidaemia	2020	EU	Alynlam/Novartis	39
Aptamer	Pegaptanib(Macugen)	AMD	2004	EU	Eyetech/Pfizer	40
Vaccine	BNT162b2(Comirnaty)	COVID-19	2021	US	BioNTech/Pfizer	26
Vaccine	mRNA-1273(Spikevax)	COVID-19	2021	US	Moderna	27

AHP, acute hepatic porphyria; AMD, age-related macular degeneration; CMV, cytomegalovirus; COVID-19, coronavirus disease 2019; DMD, duchenne muscular dystrophy; FAP, familial amyloidotic polyneuropathy; FCS, familial chylomicronemia syndrome; hereditary transthyretin amyloidosis; HoFH, homozygous familial hypercholesterolemia; PH1, primary hyperoxaluria type 1; SMA, Spinal muscular atrophy.

**Table 2:** Review of siRNA-based patents issued or under consideration for Coronaviruses

Patent number	Target region	Virus	Year
CN1458281	RdRP, S protein, M protein,packaging protein	SARS-CoV	2003
CN1465584	various	SARS-CoV	2003
CN1548054	RdRP (RNA polymerase)	SARS-CoV	2003
CN1569233	RdRp, helicase, nucleoprotein N, proteolytic enzyme gene	SARS-CoV	2003
CN1569878	target sequence in the patent	SARS-CoV	2003
CN1590545	RdRp, S protein, M gene, UP4, UP5, N	SARS-CoV	2003
CN1609116	replicase polymerase guide region	SARS-CoV	2003
CN1609117	replicase polymerase guide region	SARS-CoV	2003
US20030224353	ORF1	SARS-CoV	2003
CN1648249	M, N, E gene	SARS-CoV	2004
US20050020525	various	SARS-CoV	2005
JP2006238724	various	SARS-CoV	2005
WO2005019410	Nsp-1, Nsp-9, S protein	SARS-CoV	2005
CN101085986	ORF3a	SARS-CoV	2006
CN101113158	RdRP (RNA polymerase)	SARS-CoV	2006
CN101173275	M1 and M2 gene	SARS-CoV	2006
EP1482037	replicase (Pol) region	SARS-CoV	2006
US20050004063	replicase A1, S, N, M, E gene	SARS-CoV	2006
WO200409238	NC_004718	SARS-CoV	2007
US20070270360	NC_004718	SARS-CoV	2007
CN101182517	S, Nsp-9, Nsp-10, Nsp-13, E, M, N proteins	SARS-CoV	2007
JP2008253188	DNaseX	SARS-CoV	2007
CN102453712	PI4KB, PI4KA	SARS-CoV	2010
US20040192626	various	SARS-CoV	2012
US8653252	Replicase (Pol) region;pS3Xs (pGL3 with SARS sense, antisense target) and one without SARS target	SARS-CoV	2014
CN107488660	ORF3 25962 25983bp of GenBank DQ497008	SARS-CoV	2017
WO2017044507	P.L. pro, RdRp, S protein	MERS-CoV	2019
WO2005023083	various	SARS-CoV-2	2020
