# Supplementary material for: Development and application of ribonucleic acid therapy strategies against COVID-19
Source: Int J Biol Sci. 2022 Aug 1;18(13):5070–85. doi: 10.7150/ijbs.72706 (PMC9379410; doi:10.7150/ijbs.72706)
Supplement: Supplementary file 1 — Supplementary table 1. [file ijbsv18p5070s1.pdf]

Alias	Target regions	Sequence of siRNA (guide)	References
n7	Genes encoding nucleocapsid phosphoprotein and the surface glycoprotein	UUUGUAUGCGUCAAUAUGCUU	Chowdhury UF, Sharif Shohan MU, Hoque KI, Beg MA, Sharif Siam MK, Moni MA. A computational approach to design potential siRNA molecules as a prospective tool for silencing nucleocapsid phosphoprotein and surface glycoprotein gene of SARS-CoV-2. Genomics. 2021; 113: 331-43
g15		UCAACGUACACUUUGUUUCUG	
g21		AAAAACUUCACCAAAAGGGCA	
g22		UUAAAAACUUCACCAAAAGGG	
g44		UUAAAGCACGGUUUAAUUGUG	
g46		AACUUCUUGGGUGUUUUUGUC	
g59		UUUGAUUGUCCAAGUACACAC	
g70		UAAUUUGACUCCUUUGAGCAC	
/	Nucleocapsid gene	AGUAGAAAUACCAUCUUGGAC	Bappy SS, Shibly AZ, Sultana S, Mohiuddin AKM, Kabir Y. Designing potential siRNA molecule for the nucleocapsid(N) gene silencing of different SARS-CoV-2 strains of Bangladesh: Computational approach. Comput Biol Chem. 2021; 92: 107486.
/		UUUCUUAGUGACAGUUUGGCC	
/		ACAUUGUAUGCUUUAGUGGCA	
/		AAUUUGCGGCCAAUGUUUGUA	
/	Conserved leader sequence at the 5'end of viral genome	UCGAAAGUUGGUUGGUUUGUU	Pandey AK, Verma S. An in silico analysis of effective siRNAs against COVID-19 by targeting the leader sequence of SARS-CoV-2. Adv Cell Gene Ther. 2021: e107.
/		AUCUACAAGAGAUCGAAAGUU	
/		UUAGAGAACAGAUCUACAAGA	
/		GUUUAGAGAACAGAUCUACAA	
s1	Open-reading frame 1ab (ORF1ab) gene	UCAAUAGUCUGAACAACUGGU	Hasan M, Ashik AI, Chowdhury MB, Tasnim AT, Nishat ZS, Hossain T, et al. Computational prediction of potential siRNA and human miRNA sequences to silence orf1ab associated genes for future therapeutics against SARS-CoV-2. Inform Med Unlocked. 2021; 24: 100569.
s2		UACCUUUUUAGCUUCUUCCAC	
s3		UGUUUAGCAAGAUUGUGUCCG	
s4		UUAAAACACCCUCUUGAACAA	
s5		UAGUAGAUACACAAACACCAG	
s6		UGUUAAGUACCCAUCUACCAC	
s7		UGUAACUGGACACAUUGAGCC	
s8		UUCAUGUUGGUAGUUAGAGAA	
s9		UCUCUAUCAGACAUUAUGCAA	
s10		AUAGAUGUCAACUCAAAGCCA	
/	RNA-dependent RNA polymerase (RDRP) gene	UAGUACUACAGAUAGAGACAC	Shawan M, Sharma AR, Bhattacharya M, Mallik B, Akhter F, Shakil MS, et al. Designing an effective therapeutic siRNA to silence RdRp gene of SARS-CoV-2. Infect Genet Evol. 2021; 93: 104951.
/		UUCAUUUGAGUUAUAGUAGGG	
/		UUAAAGUUCUUUAUGCUAGCC	
/		UAAUUCUAAGCAUGUUAGGCA	
/		AUAAUUCUAAGCAUGUUAGGC	
/		UAAAAGUGCAUUAACAUUGGC	
/		AAAUAAGGCAUAUAAUUAGUA	
/	Unstructured conserved	UAAGGCAUAUAAUUAGUACAA	Rohani N, Ahmadi Moughari F, Eslahchi C. DisCoVering potential

/	regions(UCRs):ORF1ab,nsp3,n	UAUAAGGUGAAAUAAAGACAG	candidates of RNAi-based therapy for COVID-19 using computational
/	sp4,nsp14 and spike protein	AGAGGUUUGUGGUGGUUGGUA	methods. PeerJ. 2021; 9: e10505.
/		UGUAAAUUUGUUUGACUUGUG	
